# Speech rate and associations in predictive sentence processing

**DOI:** 10.3758/s13414-025-03160-0

**Published:** 2025-11-25

**Authors:** Anuenue Kukona

**Affiliations:** University of Greenwich, Old Royal Naval College, Park Row, London, SE10 9LS UK

**Keywords:** Associations, Mouse cursor tracking, Prediction, Sentence processing, Speech rate

## Abstract

Do comprehenders predict (i.e., what will come next) when hearing rapid speech? Two mouse cursor tracking experiments investigated association-based predictions, which may be suited to speeded processing. Participants heard predictive sentences (e.g., “What the pilot will fly, which is shown here, is the . . .”) while viewing visual arrays with predictable objects (e.g., helicopter) and unpredictable but verb-associated objects (e.g., kite) or unrelated objects (e.g., book). Experiment 1 compared predictive and nonpredictive (e.g., “What everyone will discuss, which is shown here, is the . . .”) sentences at a normal speech rate, and Experiment 2 compared predictive sentences at a normal and fast speech rate (e.g., averaging ~4 and 9 syllables per second). In addition to making mouse cursor movements to predictable objects before hearing predictable words (e.g., “helicopter”), participants’ mouse cursor movements at both speech rates were attracted to unpredictable but verb-associated objects, providing evidence of association-based prediction. These results suggest that when hearing rapid speech, associations support but do not dominate comprehenders’ predictions.

## Introduction

Prediction continues to attract significant attention in psycholinguistics. Not only do language comprehenders process language as it comes (i.e., bottom-up), but they also predict what will come next (i.e., top-down). Thus, prediction may be an essential feature of language comprehension. However, comprehenders’ ability to predict when hearing rapid speech has been questioned. The aim of this research was to advance understanding of prediction by investigating associations, which may be suited to speeded processing.

Predictive sentence processing is supported by significant evidence from the literature, including research using the visual world paradigm (e.g., Tanenhaus et al., 1995). For example, Altmann and Kamide’s (1999) participants heard predictive sentences like “The boy will eat the...” while viewing visual scenes with predictable objects like a cake and other unrelated objects, and these participants fixated the cake before hearing “cake” (e.g., vs. hearing “The boy will move...”). These findings suggest that comprehenders preactivate (e.g., verb-related) representations during sentence processing.

Multiple mechanisms are hypothesised to support predictive sentence processing (e.g., Huettig, 2015; Pickering & Gambi, 2018). One hypothesised mechanism emphasises associations. Prediction-by-association, to adopt Pickering and Gambi’s (2018) terminology, is hypothesised to stem from spreading activation among associated representations in memory, such that the processing of earlier words in a sentence may activate associated representations that are relevant to later words. For example, participants’ fixations to the cake when hearing “The boy will eat...” (Altmann & Kamide, 1999) may stem from activation spreading from “eat” to associated (e.g., edible) representations like cake.

Prediction-by-association is supported by evidence for “dumb” predictions. For example, Kukona et al.’s (2011) participants heard predictive sentences like “Toby will arrest the...” while viewing visual arrays with predictable objects like a crook, unpredictable but verb-associated objects like a policeman and other unrelated objects, and these participants fixated the policeman more than unrelated objects. Relatedly, Kukona et al.’s (2014) participants heard predictive sentences like “The boy will eat the white...” while viewing visual arrays with predictable objects like a white cake, unpredictable but colour-associated objects like a white car and other unrelated objects, and these participants fixated the white car more than unrelated objects (also see Kukona et al., 2016; Langlois et al., 2024; Nozari et al., 2016; Prystauka et al., 2024; Stone et al., 2021). Finally, Corps et al.’s (2022) participants heard predictive sentences produced by a female speaker like “I would like to wear the nice...” while viewing visual arrays with predictable (e.g., stereotypically feminine) objects like a dress, unpredictable (e.g., stereotypically masculine) but verb-associated objects like a tie and other unrelated objects, and these participants fixated the tie more than unrelated objects (also see Corps et al., 2023a, b). Collectively, these findings suggest that alongside preactivating predictable representations (e.g., crook when hearing “Toby will arrest the...”), comprehenders preactivate lexically associated representations that are otherwise unpredictable in sentences. Consistent with prediction-by-association, these findings suggest that activation spreads to unpredictable but associated representations (e.g., from “arrest” to policeman, “white” to white car and “wear” to tie), supporting participants’ association-based predictions. However, prediction-by-association may also be a “dumb” mechanism (e.g., see Huettig, 2015) that preactivates predictable alongside unpredictable representations.

A feature of prediction-by-association may be its effectiveness at speed. The masked priming literature emphasises the impressive speed with which associated representations interact (e.g., for review see Van den Bussche et al., 2009). For example, Kiefer’s (2002; also see Kiefer & Spitzer, 2000) participants were presented a masked prime word for 33.5 ms and then made a lexical decision to a semantically related (e.g., hen–egg) or unrelated (e.g., car–leaf) target word after a 67 ms SOA, and despite this rapidity, these participants had faster reaction times, improved accuracies and attenuated ERP responses for related compared to unrelated target words. These findings suggest that activation spreads rapidly (e.g., within tens rather than hundreds of ms) among associated representations. If prediction-by-association leverages the rapid interactivity of associated representations, then it may be suited to the processing of rapid speech, which minimises the time for processing (e.g., before what comes next does come). However, we return to this hypothesis in the General Discussion.

Helpfully, the sentence processing literature has begun to address the relationship between prediction and speech rate. Perhaps unsurprisingly, predictive sentence processing is not without temporal limits. For example, Huettig and Guerra’s (2019) participants heard predictive sentences in Dutch like “Kijk naar de afgebeelde...” (“Look at the pictured...”) while viewing visual arrays with predictable objects (e.g., consistent with the determiner “de”) like bike and other unrelated objects, and these participants fixated the bike more than unrelated objects at a slow but not normal speech rate following a one second visual preview. However, closely related research, which measured mouse cursor movements rather than fixations (i.e., while participants heard sentences and viewed visual arrays, mirroring the visual world paradigm; e.g., see Spivey et al., 2005), suggests that comprehenders preactivate representations even when hearing impressively rapid speech. Kukona’s (2023) participants heard predictive sentences like “What the man will ride, which is shown on this page, is the...” at an average speech rate of up to ~9 syllables per second while viewing visual arrays with predictable objects like a bike and other unrelated objects, and these participants made mouse cursor movements to the bike before hearing “bike” (e.g., vs. hearing “What the man will spot, which is shown on this page, is the...”). Not dissimilar to Huettig and Guerra (2019), a negative relationship was also found between prediction and speech rate.

In summary, predictive sentence processing is widely documented in psycholinguistics. Prediction has even been documented at speech rates reflecting real-world extremes (e.g., Kukona, 2023). However, whether associations support prediction when comprehenders hear rapid speech is unclear. For example, the findings from Huettig and Guerra (2019) and Kukona (2023) may stem from prediction-by-association (e.g., activation spreading from “ride” to bike in the latter) and/or a mechanism that (i.e., accurately) preactivates predictable but not unpredictable representations. Helpfully, experimental designs focused on “dumb” predictions (e.g., Kukona et al., 2011, 2014) provide an approach for investigating associations and isolating prediction-by-association.

**Fig. 1:**
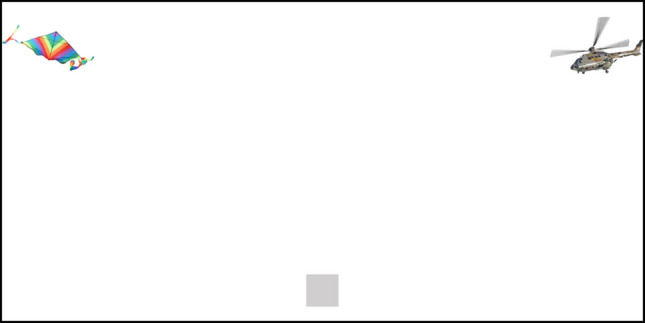
Example visual array for the predictive sentence “What the pilot will fly, which is shown here, is the helicopter.” with a predictable helicopter (i.e., target object) and unpredictable but verb-associated kite (i.e., competitor object)

The aim of this research was to investigate whether the comprehension of rapid speech is supported by association-based predictions. Building on prior research (e.g., Kukona, 2023, 2025; Kukona & Hasshim, 2024; Schlenter & Westergaard, 2024; Ye & Qu, 2025), two experiments tested for prediction by measuring participants’ mouse cursor movements. Participants heard predictive sentences like “What the pilot will fly, which is shown here, is the...” while viewing visual arrays with predictable objects like a helicopter and unpredictable but verb-associated objects like a kite or unrelated objects like a book (see Fig. 1). Predictable objects were related to both the noun (e.g., “pilot”) and verb (e.g., “fly”), unpredictable but verb-associated objects were related to the verb but not noun and unrelated objects were related to neither the noun nor verb. Experiment 1 compared predictive sentences to nonpredictive sentences (e.g., “What everyone will discuss, which is shown here, is the...”) at a normal speech rate. Experiment 2 compared predictive sentences at a normal speech rate to a fast speech rate (e.g., averaging ~4 and 9 syllables per second). Attraction to unpredictable but verb-associated objects before the predictable word (e.g., “helicopter”) provided an index of participants’ association-based predictions. If predictive sentence processing is supported by prediction-by-association, particularly at speed, then participants’ predictive mouse cursor movements (i.e., to predictable objects) were expected to be attracted to unpredictable but verb-associated objects (e.g., kite when hearing “the pilot will fly...”), and this attraction was expected to be pronounced at a fast speech rate.

## Experiment 1

Experiment 1 tested for association-based predictions by measuring mouse cursor movements to unpredictable but verb-associated objects (e.g., kite) when participants heard predictive sentences (e.g., “the pilot will fly...”) at a normal speech rate.

### Method

#### Participants

Fifty-two native speakers of English from the USA (age *M* = 39.54, *SD* = 12.47; 32 women, 20 men) were recruited to participate through Prolific (https://www.prolific.com). The sample size was adequate for detecting an average pairwise psychological effect size (e.g., Brysbaert, 2019).

#### Design and materials

Object type (competitor and distractor object) and sentence type (predictive and nonpredictive sentence) were manipulated within participants. Thirty-two object sets were created using visual stimuli from the Bank of Standardized Stimuli (Brodeur et al., 2010, 2014). Each set included a target (e.g., helicopter), competitor (e.g., kite) and distractor (e.g., book) object, as well as a predictive (e.g., “What the pilot will fly, which is shown here, is the helicopter.”) and nonpredictive (e.g. “What everyone will discuss, which is shown here, is the helicopter.”) sentence. In predictive sentences, target objects were related to both the noun and verb. In contrast, both competitor and distractor objects were unrelated to the noun, while competitor but not distractor objects were related to the verb. Latent semantic analysis (e.g., Landauer & Dumais, 1997) confirmed (i.e., among words with cosines) that the noun in predictive sentences was more related to target objects (*M* = 0.29, *SD* = 0.26) than either competitor (*M* = 0.07, *SD* = 0.12), *t*(29) = 4.30, *p* <.001, or distractor (*M* = 0.05, *SD* = 0.04), *t*(29) = 5.06, *p* <.001, objects. In addition, the verb in predictive sentences was less related to distractor objects (*M* = 0.10, *SD* = 0.08) than either target (*M* = 0.28, *SD* = 0.21), t(30) = −4.74, *p* <.001, or competitor (*M* = 0.27, *SD* = 0.17), *t*(30) = −4.56, *p* <.001, objects. Sentences were recorded using a synthesised text-to-speech Neural2 voice. To control for (e.g., visual) confounds, objects were used as both competitors and distractors across sets.

Four counterbalanced lists were created that each presented the 32 target and 32 nontarget objects once. On each list, one half of target objects were presented with a competitor object and the other half with a distractor object, while one half of target objects were presented with a predictive sentence and the other half with a nonpredictive sentence. The materials are reported in the appendix.

#### Procedure

The experiment was created in PsychoPy (e.g., Peirce et al., 2019) and internet-mediated data collection was through Pavlovia (https://pavlovia.org). Participants were presented 32 experimental trials without practice. The procedure closely followed Kukona (2023). Participants clicked on an icon at the bottom of the screen to begin each trial, they viewed a visual array like Fig. 1 with a target and nontarget (i.e., competitor or distractor) object at the top of the screen, they heard a predictive or nonpredictive sentence after a 0.50 s (i.e., visual) preview and they were instructed to click on the (i.e., target) object referred to in the sentence to end each trial. Visual arrays used normalised coordinates ranging from −1 to 1, the icon to begin was at (0, −0.85) and objects were sized 0.30 × 0.60 and centred at (±0.85, 0.70). Trial order and object location were randomised.

### Results

Four participants were excluded from the analysis: the data from one was sampled below 30 Hz, the accuracy for one was below 80% and two used a touchscreen. The overall accuracy was 99.93% (*SD* = 0.45). Inaccurate trials and trials with log RTs more than 2.50 standard deviations above the global mean were also excluded from the analysis (1.11%). Horizontal (i.e., *x*) coordinates were standardised across trials by inverting the horizontal axis when target objects were presented on the left. Coordinates of zero were at the centre of the screen, positive coordinates were toward the target object and negative coordinates were toward the nontarget object.

**Fig. 2:**
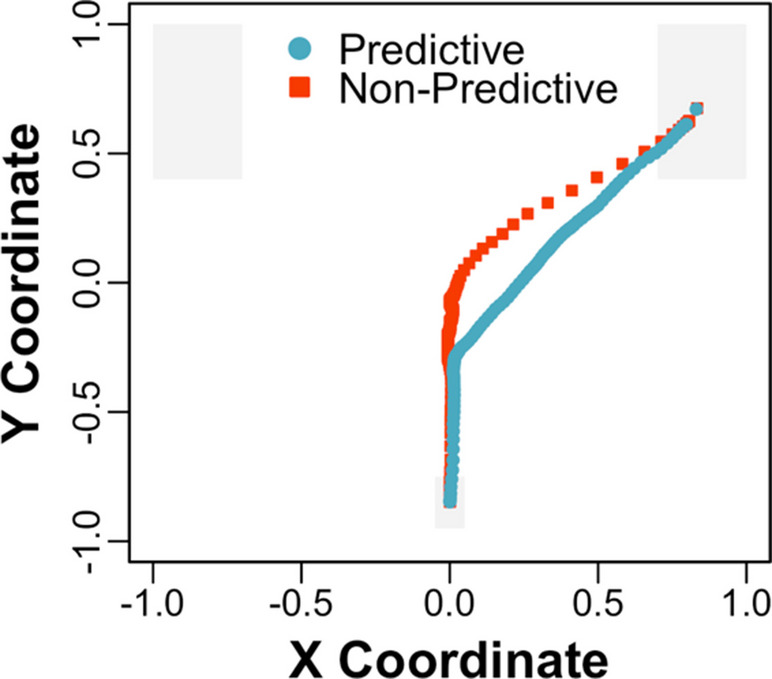
Experiment 1: Time-normalised mean trajectories across the visual array to target objects (e.g., helicopter) for predictive and nonpredictive sentences (e.g., “the pilot will fly...” vs. “everyone will discuss...”) at a normal speech rate, aggregated across object type (i.e., competitor and distractor objects)

**Fig. 3:**
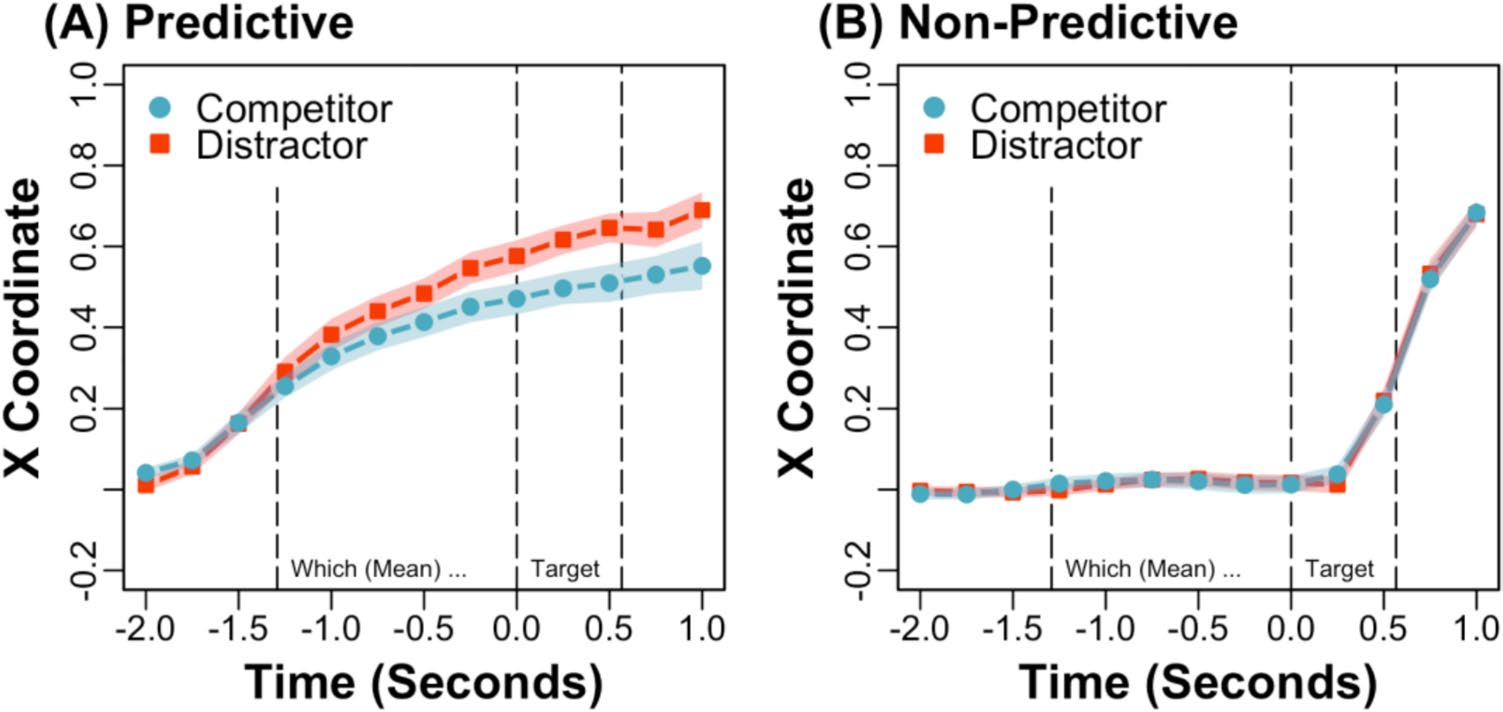
Experiment 1: Mean (shaded bands show *SEs*) horizontal *x* coordinates across time with unpredictable but verb-associated competitor (e.g., kite) and unrelated distractor (e.g., book) objects for predictive **(A)** and nonpredictive **(B)** sentences at a normal speech rate

Time-normalised mean trajectories across the visual array to target objects are depicted for predictive and nonpredictive sentences in Fig. 2, aggregated across object type (i.e., competitor and distractor objects). These normalised trajectories were generated by dividing trials into 101 time slices and aggregating the time slices across trials (e.g., see Spivey et al., 2005). Because time-normalisation obscures the time course, a simplified (e.g., aggregated) figure is presented. In addition, mean *x* coordinates across time from 2 s before target word (e.g., “helicopter”) onset to 1 s afterward are depicted with competitor and distractor objects for predictive and nonpredictive sentences in Figs. 3A and B, respectively. One additional trial was excluded from the latter figures, in which a response was made before the depicted time window.

The analysis of trajectories focused on *x* coordinates from 1 s before up to target word onset (e.g., corresponding approximately to “which is shown here, is the...”), which was before the target word (e.g., “helicopter”) but after the noun and verb (e.g., “the pilot will fly...”). Trial-level mean predictive *x* coordinates were computed by aggregating across this time window. Thus, each trial yielded a single (i.e., mean) predictive *x* coordinate (e.g., vs. multiple time points within a trial), and time was not included (e.g., as a fixed effect) in the analysis. Fifty-four additional trials were excluded from the analysis, in which a response was made before this time window (3.52%).

Following the preregistration plan, predictive *x* coordinates were submitted to a mixed-effects model with deviation-coded fixed effects of object type (competitor = −0.50, distractor = 0.50) and sentence type (predictive = −0.50, nonpredictive = 0.50) and their interaction, and (i.e., maximal) random intercepts and slopes by participants and items. Models were run in R using *lme4* (Bates et al., 2015) and *lmerTest* (Kuznetsova et al., 2017), and their random effects were simplified when there were issues with fit. The analysis revealed a significant interaction between object type and sentence type, *Est*. = −0.08, *SE* = 0.04, *t*(1383.46) = −2.29, *p* <.05. In addition, predictive *x* coordinates were submitted to (i.e., pairwise) mixed-effects models with deviation-coded fixed effects of object type for predictive and nonpredictive sentences separately. The pairwise analysis of predictive sentences revealed a significant intercept, *Est*. = 0.45, *SE* = 0.04, *t*(57.68) = 12.60, *p* <.001, such that trajectories were more attracted to target than nontarget objects overall, and a significant effect of object type, *Est*. = 0.09, *SE* = 0.03, *t*(27.49) = 2.85, *p* <.01, such that trajectories were more deflected toward competitor (*M* = 0.42, *SD* = 0.24) than distractor (*M* = 0.50, *SD* = 0.25) objects. In contrast, the pairwise analysis of nonpredictive sentences revealed a nonsignificant intercept, *Est*. = 0.02, *SE* = 0.01, *t*(29.10) = 1.28, *p* =.21, such that trajectories were not more attracted to target than nontarget objects overall, and a nonsignificant effect of object type, *Est*. = 0.00, *SE* = 0.02, *t*(735.93) = −0.01, *p* = 0.99, such that trajectories did not differ with competitor (*M* = 0.02, *SD* = 0.13) and distractor (*M* = 0.02, *SD* = 0.11) objects.

Finally, reaction times from the onset of which (e.g., which accounted for variability in noun and verb durations) were also analysed. Log reaction times were submitted to similar mixed-effects models as predictive *x* coordinates. The analysis revealed a significant interaction between object type and sentence type, *Est*. = 0.05, *SE* = 0.02, *t*(1362.11) = 2.02, *p* <.05. The pairwise analysis of predictive sentences revealed a significant effect of object type, *Est*. = −0.07, *SE* = 0.03, *t*(31.41) = −2.04, *p* <.05, such that reaction times were slower with competitor (*M* = 2.09, *SD* = 0.57) than distractor (*M* = 1.96, *SD* = 0.53) objects. In contrast, the pairwise analysis of nonpredictive sentences revealed a nonsignificant effect of object type, *Est*. = −0.01, *SE* = 0.01, *t*(685.25) = −1.29, *p* =.20, such that reaction times did not differ with competitor (*M* = 2.51, *SD* = 0.35) and distractor (*M* = 2.48, *SD* = 0.37) objects.

### Discussion

In Experiment 1, participants heard predictive sentences like “What the pilot will fly, which is shown here, is the...”, and they made mouse cursor movements to predictable objects like a helicopter before hearing “helicopter”. These results suggest that mouse cursor movements are sensitive to predictive sentence processing, conceptually replicating Kukona (2023). In addition, participants’ predictive mouse cursor movements were more attracted to unpredictable but verb-associated objects like a kite than unrelated objects like a book (see Fig. 3A). These results suggest that mouse cursor movements are sensitive to association-based predictions (e.g., Kukona et al., 2011, 2014). In contrast, these (e.g., predictive) mouse cursor movements were not observed when participants heard nonpredictive sentences like “What everyone will discuss, which is shown here, is the...”, and their reaction times were also slower (e.g., see the significant interactions between object type and sentence type). Consistent with prediction-by-association, these results suggest that activation spread from “fly” to kite, supporting participants’ association-based predictions. In Experiment 1, sentences were presented at a normal speech rate. In Experiment 2, sentences were presented at a normal or fast speech rate to address the relationship between speech rate and association-based predictions.

## Experiment 2

Experiment 2 addressed the relationship between speech rate and association-based predictions by measuring mouse cursor movements to unpredictable but verb-associated objects (e.g., kite) when participants heard predictive sentences (e.g., “the pilot will fly...”) at a normal or fast speech rate.

### Method

#### Participants

Fifty-two participants (age *M* = 34.58, *SD* = 10.39; 27 women, 26 men) were recruited using the same criteria as Experiment 1.

#### Design and materials

Object type (competitor and distractor object) and (i.e., speech) rate type (normal and fast rate) were manipulated within participants. The 32 object sets from Experiment 1 were modified to include predictive sentences (e.g., “What the pilot will fly, which is shown here, is the helicopter.”) at a normal and fast speech rate. For the fast rate, the durations of the predictive sentences from Experiment 1 (i.e., at a normal rate) were halved (i.e., their speech rates were doubled) using the duration manipulation function in Praat (Boersma, 2001). The mean speech rate was 4.44 (*SD* = 0.37) syllables per second at the normal rate and 8.88 (*SD* = 0.73) syllables per second at the fast fate.

Four counterbalanced lists were created that each presented the 32 target and 32 nontarget objects once. On each list, one half of target objects were presented with a competitor object and the other half with a distractor object, while one half of target objects were presented with a predictive sentence at a normal rate and the other half at a fast rate.

#### Procedure

The procedure was identical to Experiment 1, except that participants heard a predictive sentence at a normal or fast rate on each trial and they did not hear nonpredictive sentences.

### Results

**Fig. 4:**
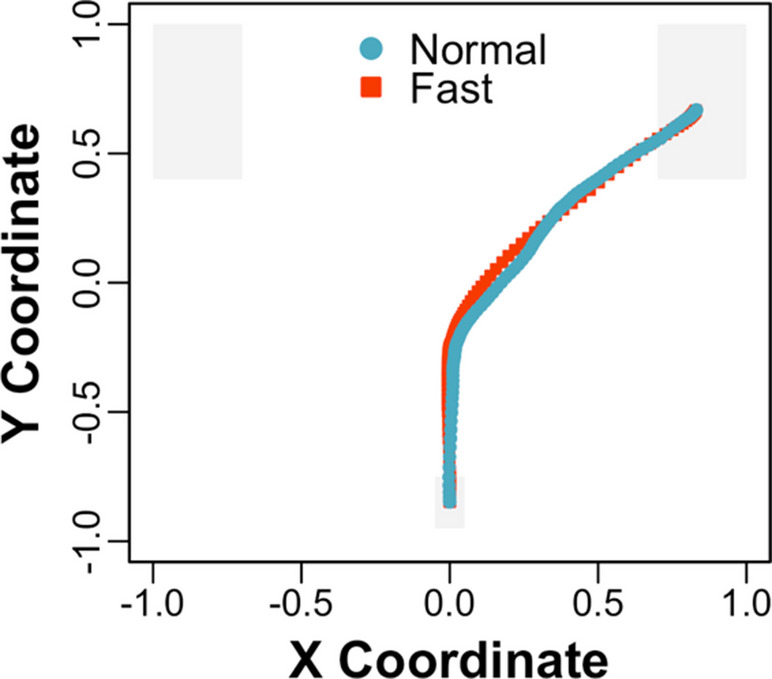
Experiment 2: Time-normalised mean trajectories across the visual array to target objects for predictive sentences at a normal and fast speech rate (e.g., averaging ~4 vs. 9 syllables per second), aggregated across object type (i.e., competitor and distractor objects)

**Fig. 5:**
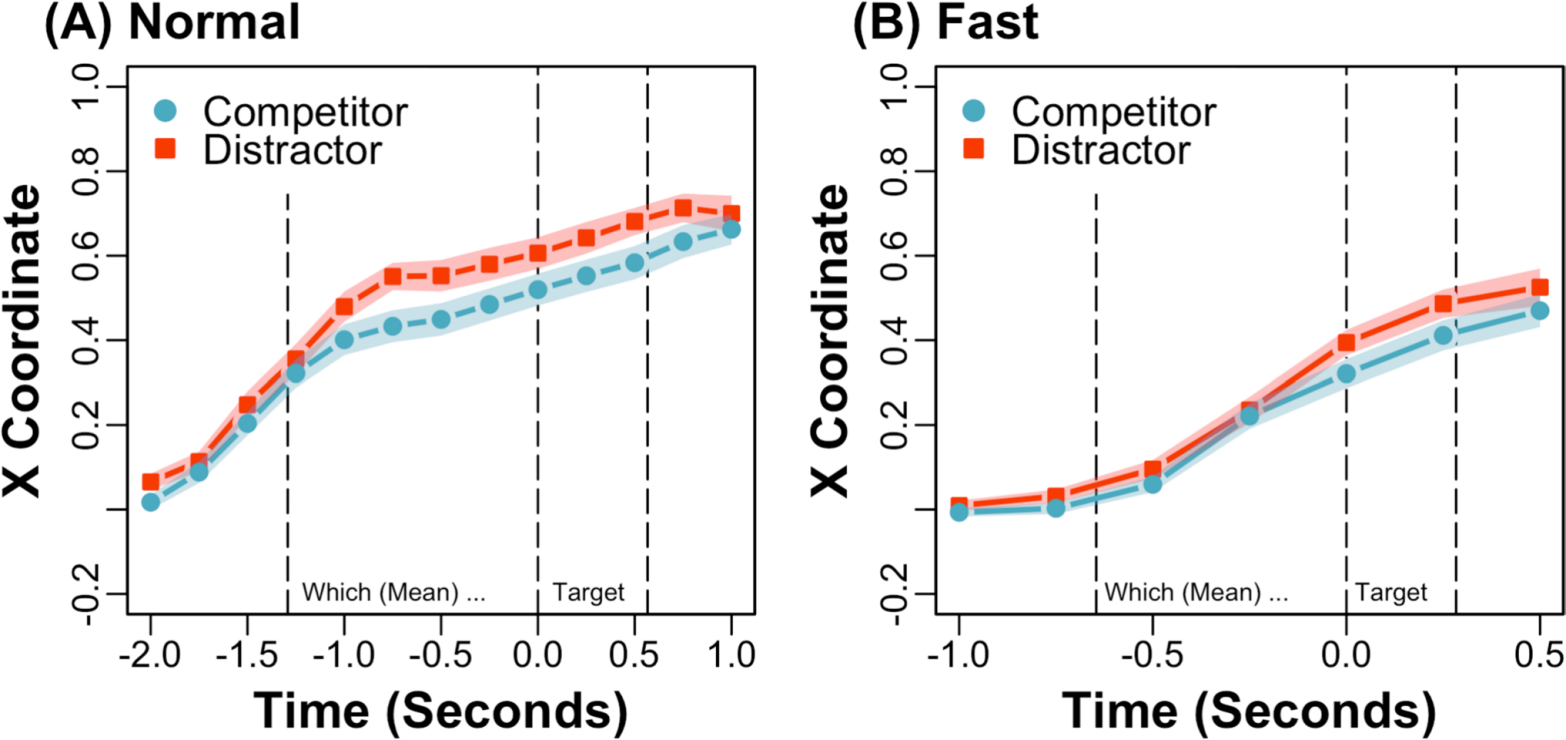
Experiment 2: Mean (shaded bands show *SEs*) horizontal *x* coordinates across time with unpredictable but verb-associated competitor and unrelated distractor objects for predictive sentences at a normal **(A)** and fast **(B)** speech rate

Five participants were excluded from the analysis: the data from two was sampled below 30 Hz and the accuracy for three was below 80%. The overall accuracy was 99.20% (*SD* = 2.56). Inaccurate trials and trials with log RTs more than 2.50 standard deviations above the global mean by rate type were also excluded from the analysis (2.39%). Time-normalised mean trajectories across the visual array to target objects are depicted for predictive sentences at a normal and fast rate in Fig. 4, aggregated across object type (i.e., competitor and distractor objects). In addition, mean *x* coordinates across time are depicted with competitor and distractor objects for predictive sentences at a normal and fast rate in Fig. 5A and B, respectively. The time window spans 2 s before target word onset to 1 s afterward at the normal rate, and 1 s before target word onset to 0.50 s afterward at the fast rate, which contain equivalent linguistic content.

The analysis of trajectories focused on (i.e., predictive) *x* coordinates from 1 s before up to target word onset for the normal rate and from 0.5 s before up to target word onset for the fast rate, which contain equivalent linguistic content (e.g., corresponding approximately to “which is shown here, is the...”). One hundred five additional trials were excluded from the analysis, in which a response was made before this time window (6.98%). Following the preregistration plan, predictive *x* coordinates were submitted to a mixed-effects model with deviation-coded fixed effects of object type (competitor = −0.50, distractor = 0.50) and rate type (normal = −0.50, fast = 0.50) and their interaction. The analysis revealed a significant interaction between object type and rate type, *Est*. = −0.07, *SE* = 0.03, *t*(1225.27) = −2.21, *p* <.05. In addition, predictive *x* coordinates were submitted to (i.e., pairwise) mixed-effects models with deviation-coded fixed effects of object type for a normal and fast rate separately. The pairwise analysis of the normal rate revealed a significant intercept, *Est*. = 0.51, *SE* = 0.03, *t*(56.02) = 16.18, *p* <.001, such that trajectories were more attracted to target than nontarget objects overall, and a significant effect of object type, *Est*. = 0.11, *SE* = 0.03, *t*(30.40) = 3.87, *p* <.001, such that trajectories were more deflected toward competitor (*M* = 0.48, *SD* = 0.24) than distractor (*M* = 0.58, *SD* = 0.21) objects. Similarly, the pairwise analysis of the fast rate revealed a significant intercept, *Est*. = 0.22, *SE* = 0.03, *t*(56.42) = 7.37, *p* <.001, such that trajectories were more attracted to target than nontarget objects overall, and a significant effect of object type, *Est*. = 0.05, *SE* = 0.02, *t*(679.66) = 2.23, *p* <.05, such that trajectories were more deflected toward competitor (*M* = 0.21, *SD* = 0.17) than distractor (*M* = 0.25, *SD* = 0.18) objects. The significant interaction (i.e., in the model with both object type and rate type) also suggests that deflections toward competitor objects were greater at a normal (e.g., 0.58 distractor − 0.48 competitor = 0.10) than fast (e.g., 0.25 distractor − 0.21 competitor = 0.04) rate.

As an additional exploratory analysis, competition scores were computed, which captured differences in participants’ trajectories with competitor vs. distractor objects. Participant-level competition scores were computed by aggregating across trials and subtracting (i.e., mean) predictive *x* coordinates with competitors objects from distractor objects at a normal and fast rate separately. As a comparison, prediction scores were also computed, which captured participants’ mouse cursor movements to target objects with both competitor and distractor objects. Participant-level prediction scores were computed by aggregating across trials and averaging (i.e., mean) predictive *x* coordinates with competitors and distractor objects at a normal and fast rate separately. One additional participant was excluded from the analysis, who had a competition score more than 4 standard deviations below the mean. Scores were submitted to mixed-effects models with deviation-coded fixed effects of rate type. The analysis of competition scores revealed a significant effect of rate type, *Est*. = −0.08, *SE* = 0.03, *t*(45) = −2.37, *p* <.05, such that attraction to competitor objects was greater at a normal (*M* = 0.12, *SD* = 0.19) than fast (*M* = 0.04, *SD* = 0.14) rate. Similarly, the analysis of prediction scores revealed a significant effect of rate type, *Est*. = −0.30, *SE* = 0.02, *t*(45) = −13.38, *p* <.001, such that mouse cursor movements to target objects were greater at a normal (*M* = 0.53, *SD* = 0.19) than fast (*M* = 0.23, *SD* = 0.16) rate. In addition, scores were submitted to a mixed-effects model with deviation-coded fixed effects of rate type and score type (competition score = −0.50, prediction score = 0.50) and their interaction, which revealed a significant interaction between rate type and score type, *Est*. = −0.22, *SE* = 0.05, *t*(135) = −4.61, *p* <.001. The significant interaction suggests that participants’ attraction to competitor objects (e.g., 0.12 normal − 0.04 fast = 0.08) was less affected by speech rate than their mouse cursor movements to target objects (e.g., 0.53 normal − 0.23 fast = 0.30).

Finally, reaction times were also analysed. The analysis revealed a nonsignificant interaction between object type and rate type, *Est*. = 0.02, *SE* = 0.04, *t*(1197.20) = 0.49, *p* =.62, but a significant main effect of object type, *Est*. = −0.08, *SE* = 0.02, *t*(21.07) = −3.33, *p* <.01, such that reaction times were slower with competitor (normal rate: *M* = 2.08, *SD* = 0.63; fast rate: *M* = 1.51, *SD* = 0.43) than distractor (normal rate: *M* = 1.88, *SD* = 0.63; fast rate: *M* = 1.43, *SD* = 0.40) objects, and a significant main effect of rate type, *Est*. = −0.26, *SE* = 0.04, *t*(41.66) = −7.11, *p* <.001, such that reaction times were slower at a normal rate than fast rate.

### Discussion

In Experiment 2, participants heard predictive sentences like “What the pilot will fly, which is shown here, is the...”, and they made mouse cursor movements to predictable objects like a helicopter before hearing “helicopter” at both a normal and fast speech rate. These results suggest that comprehenders preactivate representations even when hearing impressively rapid speech (e.g., averaging ~9 syllables per second), conceptually replicating Kukona (2023). In addition, participants’ predictive mouse cursor movements were more attracted to unpredictable but verb-associated objects like a kite than unrelated objects like a book (e.g., replicating Experiment 1) at both speech rates (see Figs. 5A and B). While participants’ attraction to unpredictable but verb-associated objects was weaker at a fast speech rate, their mouse cursor movements to predictable objects were also weaker. On balance, these results suggest that association-based predictions persist, but are not more pronounced, at a rapid speech rate.

## General Discussion

Two mouse cursor tracking experiments investigated whether associations support prediction when comprehenders hear rapid speech. Participants hearing predictive sentences (e.g., “What the pilot will fly, which is shown here, is the...”) made mouse cursor movements to predictable objects (e.g., helicopter) before hearing predictable words (e.g., “helicopter”) at both a normal and fast speech rate. In addition, participants’ predictive mouse cursor movements at both speech rates were attracted to unpredictable but verb-associated objects (e.g., kite), providing evidence of association-based prediction. These results provide novel insight into the persistence of association-based predictions in sentence processing.

These results extend prior findings from the sentence processing literature. Conceptually replicating Kukona (2023), these results suggest that comprehenders preactivate representations even when hearing impressively rapid speech, which their mouse cursor movements are also sensitive to. However, in contrast to Kukona (2023), Experiment 2 manipulated speech rate within rather than between participants. Thus, these results emphasise comprehenders’ ability to cope with (i.e., speech rate) extremes as well as variability. In addition, complementing findings like Kukona et al. (2011, 2014), these results suggest that comprehenders preactivate lexically associated representations that are otherwise unpredictable in sentences. However, in contrast to prior research, Experiments 1 and 2 measured mouse cursor movements rather than fixations. Thus, these results emphasise the pervasive effects of association-based predictions on behaviour. Moreover, not only were participants’ trajectories attracted to unpredictable but verb-associated objects, but their corresponding reaction times were also slowed. These results also complement recent findings from Ye and Qu (2025; e.g., alongside related semantic findings; Dale et al., 2007; Toon et al., 2023). Their participants heard constraining sentences while viewing visual arrays with predictable objects and objects that were either semantically related or unrelated to the predictable objects. They measured mouse cursor movements and found greater attraction to related than unrelated objects. However, in contrast to Ye and Qu (2025), Experiments 1 and 2 focused on verb associations rather than noun associations. Thus, these results emphasise the effects of varied associations on predictive sentence processing. Finally, these results also provide evidence of association-based prediction at a fast speech rate, which prior research has not addressed.

It was hypothesised that prediction-by-association (e.g., Pickering & Gambi, 2018; also see Huettig, 2015) may be suited to the processing of rapid speech. For example, activation spreads rapidly among associated representations (e.g., see Van den Bussche et al., 2009). If prediction-by-association leverages this rapidity, then it may be effective at speed. On the one hand, this hypothesis was supported by these results: participants’ predictive mouse cursor movements in Experiment 2 were attracted to unpredictable but verb-associated objects even at a fast speech rate. On the other hand, this attraction was weaker at a fast compared to normal speech rate, although mouse cursor movements to predictable objects were also weaker. On balance, these results suggest that when hearing rapid speech, associations support but do not dominate comprehenders’ predictions. To the contrary, participants’ predictions remained highly accurate even at a fast speech rate. Thus, these results also raise questions about the other mechanisms that may support prediction.

Pickering and Gambi (2018) hypothesise that alongside prediction-by-association, predictive sentence processing is supported by prediction-by-production. This mechanism emphasises the link between comprehension and production, such that comprehenders may use their production systems (e.g., covertly) to complete (i.e., speakers’) incomplete sentences (e.g., not unlike responding in a cloze task). Prediction-by-production complements evidence for form (e.g., vs. conceptual) predictions, which are particularly relevant to production. For example, Ito et al.’s (2018) participants heard predictive sentences like “The tourists expected rain when the sun went behind the...” while viewing visual arrays with predictable objects like a cloud, unpredictable but form-related objects like a clown and other unrelated objects, and these participants fixated the clown more than unrelated objects (also see Ito, 2024; Kukona, 2020). These findings suggest that comprehenders preactivate words (e.g., not merely concepts), which co-activate form-related cohorts (e.g., “cloud” alongside “clown”).

Prediction-by-production (e.g., Pickering & Gambi, 2018) is compatible with aspects of these results. In other words, participants may have used their production systems to complete sentences at both a normal and fast speech rate (e.g., like responding “helicopter” to “the pilot will fly _” in a cloze task). Relatedly, Pickering and Gambi (2018) emphasise the importance of timing in prediction-by-production: this mechanism is assumed to involve multiple stages, which comprehenders may be unlikely to progress through when hearing rapid speech. Thus, prediction-by-production may not be suited to the processing of rapid speech. Consistent with this hypothesis, Ito et al. (2016) found that form-based predictions, which are particularly relevant to production, were weaker at a rapid speech rate. Their participants read predictive sentences like “The student is going to the library to borrow a...” that ended in predictable words like “book”, unpredictable but semantically-related words like “page”, unpredictable but form-related words like “hook” or unrelated words like “sofa”, and these participants’ ERP responses to form-related words were weaker with a faster compared to slower presentation rate. Similarly, participants’ predictive mouse cursor movements to predictable objects in Experiment 2 were weaker at a fast compared to normal speech rate. Nevertheless, participants’ predictions remained highly accurate. In fact, properties of the materials may have allowed prediction-by-production to cope with a rapid speech rate (e.g., so that it was perhaps as suited to speeded processing as prediction-by-association). The sentences in Experiments 1 and 2 included filler words (i.e., “which is shown here”), which provided a temporal buffer (e.g., between “the pilot will fly...” and “helicopter”) and may have allowed prediction-by-production to progress through its multiple stages. In addition, the strong relationship between nouns (e.g., “pilot”) and predictable objects (e.g., helicopter) may have allowed participants’ production systems to rapidly generate sentence completions. On balance, these results are compatible with a multiple mechanisms approach to predictive sentence processing that is supported by both prediction-by-production and prediction-by-association. However, Experiments 1 and 2 did not address form-based predictions directly, reflecting an important direction for future research.

These results may also be compatible with a mechanism that engages with associations in a more sophisticated way. Prediction-by-association was assumed to involve simple excitatory spreading activation among associated representations in memory: as each word in a sentence is heard, associated representations are preactivated irrespective of earlier information. In contrast, these results suggest that prediction may rely on more complex excitation and inhibition dynamics. Relatedly, models of semantic knowledge emphasise both excitation and inhibition (e.g., Chen & Mirman, 2012; Mirman & Magnuson, 2009). Similarly, comprehenders’ predictions may stem from a mechanism in which excitatory spreading activation is (e.g., gradiently) constrained by inhibition from earlier information, such that representations activated by earlier words in a sentence may inhibit representations associated with later words. For example, representations activated by “pilot” may inhibit representations associated with “fly” like kite, but not representations associated with both “pilot” and “fly” like helicopter. Consistent with this hypothesis, participants’ predictions were not dominated by associations in Experiments 1 and 2. Moreover, this mechanism may still leverage the rapid interactivity of associated representations. For example, participants’ compelling attraction to helicopter at even a fast speech rate may stem from rapid excitatory spreading activation from “pilot”. In fact, processing at “pilot” resembled a typical semantic priming paradigm (i.e., in which there are no words before the prime; e.g., see Van den Bussche et al., 2009), so it may have benefited from the rapidness of excitatory spreading activation alongside a lack of inhibition (i.e., there were no earlier words to inhibit its associations). On balance, these results suggest that an emphasis on “dumb” predictions may be too simplistic; rather, the (e.g., excitatory and inhibitory) dynamics of association-based predictions reflect an important direction for future research.

Finally, an important limitation of these results is their focus on a narrow type of association. The associated objects in Experiments 1 and 2 depended on verb semantics (e.g., fly-kite) and conceptual relationships. In contrast, findings from the literature reflect varied associations. For example, Kukona et al. (2014) focused on adjective semantics (e.g., “The boy will eat the white...”) and visual relationships (e.g., white car). Simultaneously, the associated objects in Experiments 1 and 2 reflected varied semantic roles, including themes (e.g., “fly... the kite”), patients (e.g., “ride... the skateboard”) and instruments (e.g.., “write with... the crayon”). While verb semantics and conceptual relationships dominate the literature (e.g., Altmann & Kamide, 1999), these results do not address other association-based predictions and their interactions with speech rate directly. Thus, an important direction for future research will be to investigate richer associations. Another limitation centres on time course comparisons between normal and fast speech rates. The time window in Experiment 2 contained equivalent linguistic content at both rates (e.g., corresponding approximately to “which is shown here, is the...”), but differed temporally (e.g., at a fast rate, the time window was shorter in duration and based on fewer data samples). In addition, the analysis aggregated across time. Thus, an important direction for future research will be to closely investigate the time course of processing.

In conclusion, these experiments provide novel insight into the persistence of association-based predictions in sentence processing. Participants generated predictions based on verb associations (e.g., such that their mouse cursor movements were attracted to kite when hearing “the pilot will fly...”) even at a fast speech rate. These results suggest that associations are integral to prediction, and they support a multiple mechanisms approach to predictive sentence processing (e.g., Huettig, 2015; Pickering & Gambi, 2018). Finally, complementing prior findings (e.g., Kukona, 2023, 2025; Kukona & Hasshim, 2024; Schlenter & Westergaard, 2024; Ye & Qu, 2025), these results emphasise the fine-grained temporal and spatial sensitivity of mouse cursor tracking to the dynamics of predictive sentence processing.

## Data Availability

The data is available at OSF.

## Appendix

Table 1 reports the materials from Experiments 1 and 2. Predicative sentences referred to the noun, verb and target (e.g., “What the pilot will fly, which is shown here, is the helicopter.”). Visual arrays included the target (e.g., helicopter) and competitor (e.g., kite) or distractor (e.g., book).

**Table 1.** Materials from Experiments 1 and 2

Item	Noun	Verb	Target	Competitor	Distractor
1	Preschooler	Build	Sand Castle	Bridge	Grapes
2	Monkey	Devour	Banana	Fortune Cookie	Car
3	Toddler	Eat	Candy	Broccoli	Arrow
4	Mechanic	Kick	Tire	Soccer Ball	Broccoli
5	Hippie	Pick	Flower	Grapes	Skateboard
6	Cowboy	Ride	Horse	Skateboard	Fortune Cookie
7	Sniper	Shoot	Bullet	Arrow	Soccer Ball
8	Cook	Wash	Rice	Car	Bridge
9	Baker	Decorate	Cupcake	Christmas Tree	Binoculars
10	Mover	Drive	Truck	Tank	Christmas Tree
11	Pilot	Fly	Helicopter	Kite	Book
12	Molecular Biologist	Look Through	Microscope	Binoculars	Kite
13	Pianist	Read	Music Sheet	Book	Volleyball
14	Waiter	Serve	Sushi	Volleyball	Tank
15	Pitcher	Throw	Baseball	Paper Airplane	Apron
16	Athlete	Wear	Football Helmet	Apron	Paper Airplane
17	Pastry Chef	Bake	Croissant	Potato	Tennis Ball
18	Child	Drive	Bumper Car	Jeep	Potato
19	Boxer	Hit	Punching Bag	Tennis Ball	Keys
20	Astronomer	Look Through	Telescope	Magnifying Glass	Poker Chips
21	Freshman	Ride	School Bus	Tricycle	Dog House
22	Camper	Sleep In	Tent	Dog House	Tricycle
23	Player	Toss	Frisbee	Keys	Magnifying Glass
24	Olympian	Win	Medal	Poker Chips	Jeep
25	Vegan	Devour	Pineapple	Bacon	Spear
26	Vegetarian	Eat	Granola Bar	Shrimp	Crayon
27	Gymnast	Jump On	Trampoline	Bed	Bacon
28	Nurse	Push	Wheelchair	Lawn Mower	Bib
29	Blacksmith	Sharpen	Sword	Pencil	Shrimp
30	Quarterback	Throw	Football	Spear	Lawn Mower
31	King	Wear	Crown	Bib	Pencil
32	Philosopher	Write With	Pen	Crayon	Bed

## Code availability

The analysis code is available at OSF.

## Declarations

### Open practices statement

The data and analysis code are available at OSF (10.17605/OSF.IO/SY6JC). Experiments 1 (10.17605/OSF.IO/KMYZR) and 2 (10.17605/OSF.IO/8R4YQ) were preregistered.
